# TFCP2 Genetic Polymorphism Is Associated with Predisposition to and Transplant Prognosis of Hepatocellular Carcinoma

**DOI:** 10.1155/2017/6353248

**Published:** 2017-02-27

**Authors:** Zhikun Liu, Feng Gao, Zhou Shao, Haiyang Xie, Lin Zhou, Xiao Xu, Shusen Zheng

**Affiliations:** ^1^Division of Hepatobiliary and Pancreatic Surgery, First Affiliated Hospital, Zhejiang University School of Medicine, Hangzhou, China; ^2^Key Lab of Combined Multi-Organ Transplantation, Ministry of Public Health, Hangzhou, China; ^3^Collaborative Innovation Center for Diagnosis and Treatment of Infectious Diseases, Hangzhou, China

## Abstract

TFCP2 is an oncogene and plays crucial roles in the incidence and progression of hepatocellular carcinoma (HCC). However, no reports are available on the impact of TFCP2 genetic polymorphism on the susceptibility to and the transplant prognosis of HCC. Here, we genotyped 7 SNPs of TFCP2 in a case-control study of 119 patients with HCC and 200 patients with chronic liver disease. Of the 7 SNPs in TFCP2, rs7959378 distributed differentially between patients with versus patients without HCC. The patients with the CA (OR = 0.58, 95% CI = 0.35–0.96), the CC (OR = 0.39, 95% CI = 0.20–0.76), and the CA/CC (OR = 0.52, 95% CI = 0.32–0.83) genotypes had significantly decreased risk for HCC compared with those carrying the rs7959378 AA genotype. After adjusting for confounding factors, rs7959378 still conferred significant risk for HCC. Furthermore, the patients who carried rs7959378 AC/CC had a higher overall survival and lower relapse-free survival than those with the rs7959378 AA genotype. Similar results were found in the multivariate analysis adjusted by AFP, tumor size and tumor number, and differentiation. These findings indicate that rs7959378 is associated with the risk of HCC in patient with chronic liver disease and prognosis of HCC patients after liver transplantation.

## 1. Introduction

Hepatocellular carcinoma (HCC) is a worldwide prevalent and deadly neoplasia [1]. HCC occurs usually in the background of chronic liver diseases, such as hepatitis virus infection and liver cirrhosis [2, 3]. The prevalence of hepatitis B virus (HBV) carriage is reported to be 350 million people worldwide [4]. In China, chronic HBV infection has a high prevalence with approximately 93 million individuals (National Health and Family Planning Commission of the PRC). In addition to HBV, chronic infection with the hepatitis C virus, excessive alcohol consumption, and heavy aflatoxin exposure have also been proposed as risk factors for HCC [5–7]. Some genetic markers have also been reported to be HCC risk and prognostic factors [8]. Despite those advances, as a multifactorial and complex process, the exact pathogenesis of HCC is still unclear.

The transcription factor CP2 (TFCP2) has been shown to regulate diverse cellular and viral promoters and plays roles in cell cycle progression and cell survival [9–12]. Yoo et al. [13] firstly reported that TFCP2 overexpression is detected in more than 90% of cases of human HCC patients compared to normal liver and is associated with the stage and grade of the disease. There are increasing evidences that TFCP2 is a key factor for hepatocarcinogenesis and prognosis. Additionally, TFCP2 contributes to 5-fluorouracil resistance [14]. To understand the mechanisms of TFCP2 involved in HCC development and progression, our colleagues and other groups have found that TFCP2 could enhance invasion and angiogenesis of HCC via regulating osteopontin, fibronectin 1, and matrix metalloproteinase-9, respectively [13, 15, 16]. The pivotal role of TFCP2 in hepatocarcinogenesis is alternatively confirmed by the fact that TFCP2 suppression by FQI1, a specific small molecule inhibitor of TFCP2 binding to DNA, is an effective therapeutic approach for treating HCC [17].

Genetic factors may also play critical roles in the development and prognosis of HCC. Previous studies have demonstrated that genetic variant mainly in the form of single nucleotide polymorphism (SNP) plays an important role in carcinomagenesis, tumor recurrence, and prognosis of HCC patients [18, 19]. Although a close association between TFCP2 protein and HCC has been established, the genetic polymorphism of TFCP2 gene and HCC remains unknown. Seven gene polymorphisms at different loci have been identified for TFCP2 gene, including rs10876135, rs11169735, rs1056897, rs10099, rs12820966, rs7959378, and rs11169736. In this study, we enrolled chronic liver disease with or without HCC patients to study whether the above-mentioned TFCP2 polymorphisms can affect the risk and transplant prognosis of HCC.

## 2. Material and Methods

### 2.1. Subjects

The present study included 200 patients with chronic liver diseases as control and 119 patients with primary HCC who underwent liver transplantation (LT) between January 2008 and December 2012 at the First Affiliated Hospital, Zhejiang University School of Medicine, China. The diagnosis was confirmed by pathological examination and the recurrence by AFP elevation (>400 ng/ml) and/or imaging examination (MRI/CT). The subjects' recruitment was approved by the Institutional Review Board of the First Affiliated Hospital, Zhejiang University, according to the Declaration of Helsinki. Written informed consents were obtained. Data were analyzed anonymously.

All patients received LT as the initial therapy. The transarterial chemoembolization (TACE) and radiofrequency ablation (RFA) procedure were performed as the postoperative therapy in the patients with HCC relapse, if indicated. Complete follow-up data were obtained from all HCC patients (range, 1 month–66 months; median, 26 months). Primary study end points were overall survival (OS) and relapse-free survival (RFS). OS and RFS were defined as the time from the date of surgery to the date of death from HCC or to the date of local recurrence or detection of distant metastasis, respectively.

### 2.2. SNP Selection and Genotyping

Genomic DNA was isolated from EDTA-anticoagulated whole blood of recipients using the QIAamp DNA Blood mini kit (QIAGEN, Hilden, Germany). The potential functional SNPs of TFCP2 with minor allele frequency (MAF) of greater than 0.20 for the Han Chinese were selected from the entire gene region according to the HapMap database. Seven SNPs were found, namely, rs7959378 (5′ flanking), rs11169736 (exon1), rs1056897 (exon1 (5′-UTR)), rs11169735 (exon1 (5′-UTR)), rs12820966 (exon15 (3′-UTR)), rs10876135 (exon15 (3′-UTR)), and rs10099 (exon15 (3′-UTR)). The selected SNPs were detected in chronic liver disease patients with or without HCC using Applied Biosystems SNaPshot and TaqMan technology.

### 2.3. Statistical Analysis

The Hardy-Weinberg equilibrium was evaluated using Pearson's *χ*^2^ test separately for HCC subjects and controls. The TFCP2 genotype distributions and allele frequencies were compared by using *χ*^2^ analysis or Fisher's exact test. The multivariate logistic regression analysis was conducted to determine the association of TFCP2 polymorphism and HCC susceptibility, with the adjustment with several noncomparable factors, such as gender, MELD score, and Child score at enrollment. For survival comparison, we performed the Kaplan-Meier analyses stratified by the TFCP2 genotypes. The significance of the differences in survival among different genotype carriers was evaluated with the log-rank test. The univariate and multivariate Cox proportional hazard models were conducted to determine the prognostic values of TFCP2 genotype in HCC patients. The hazard ratio (HR) and the 95% confidence interval (95% CI) were calculated. All these above-mentioned statistical analyses were performed by using the SPSS software package (version 18.0; SPSS Inc., Chicago, Illinois). A *P* value less than 0.05 was considered to be statistically significant.

## 3. Results

### 3.1. Clinical Characteristics of the Study Population

A total of 319 patients were included and grouped into non-HCC group (*n* = 200) and HCC group (*n* = 119). The basic parameters of the two groups are summarized in Table 1. HBV constituted the majority of the etiology in both the non-HCC and the HCC group, and the proportion of HBV was similar between the two groups (76% versus 82%, *P* = 0.164). Compared to the non-HCC group, the HCC patients were predominantly men (*P* = 0.008) and had significantly lower MELD and Child scores (*P* < 0.001). The genotype frequencies of all SNPs in the control groups were in the Hardy-Weinberg equilibrium (all *P* values > 0.05).

### 3.2. Association Analysis of Candidate SNPs with HCC Risk

We conducted genotyping experiments for the 7 TFCP2 polymorphisms in chronic liver disease patients without HCC (*n* = 200) and with HCC (*n* = 119). The genotype frequencies of TFCP2 gene polymorphism in HCC and controls are presented in Table 2. Of the 7 SNPs in TFCP2, rs7959378 distributed differentially between patients with and without HCC. For rs7959378 polymorphism, the HCC patients had a lower prevalence of CC (14%) than the control subjects (23%, *P* = 0.041). For allele comparison, HCC subjects had lower C allele frequency (34%) than the controls (47%, *P* = 0.002). Compared with the patients carrying the rs7959378 AA genotype, those with the CC genotype had a decreased risk for HCC with an OR of 0.39 (95% CI 0.20–0.76, *P* = 0.006) and the CA/CC genotypes had a decreased risk for HCC (OR = 0.52, 95% CI 0.32–0.83, *P* = 0.006). After adjusting for gender, MELD score, and Child score, rs7959378 also conferred significant risk for the disease (CC genotype, OR = 0.43, 95% CI 0.20–0.96, *P* = 0.030; CA/CC genotypes, OR = 0.57, 95% CI 0.33–0.98, *P* = 0.044). We further analyzed the effect of the alleles of rs7959378. With rs7959378 A allele as reference, the *OR* for rs7959378 C allele carriage was 0.63 (95% CI 0.43–0.93, *P* = 0.018). For the other SNPs, the genotype and allele frequencies were not associated with the HCC risk after the adjustment with the aforementioned confounding factors.

### 3.3. TFCP2 rs7959378 Was Associated with the Prognosis of HCC Patients after LT

**Figure 1:**
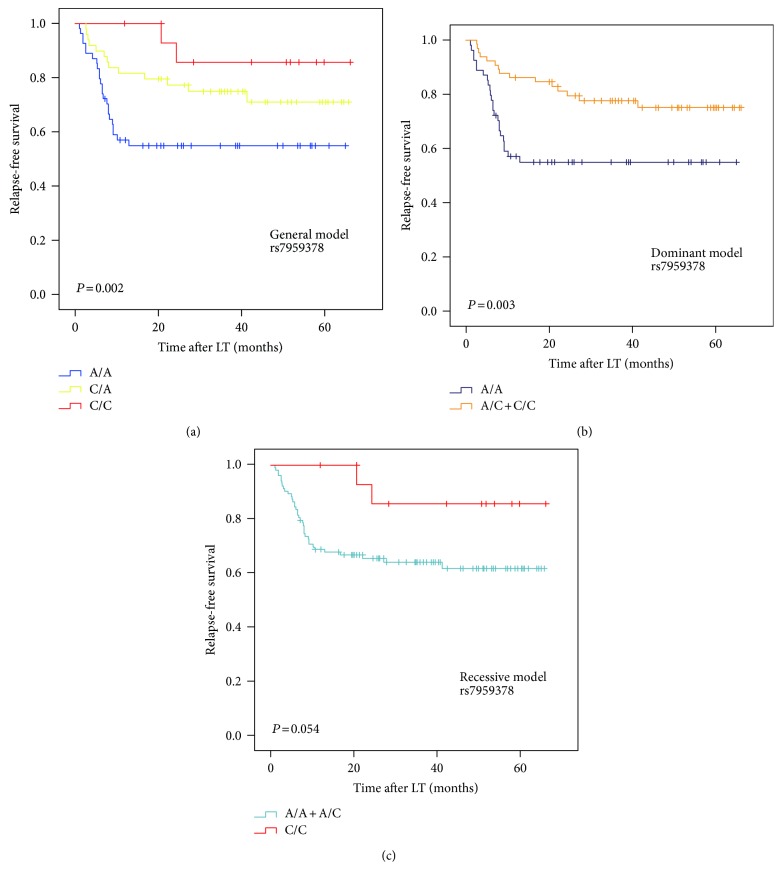
The Kaplan-Meier survival curves for relapse-free survival of the HCC patients stratified by rs7959378 genotypes under general model (a), dominant model (b), and recessive model (c).

**Figure 2:**
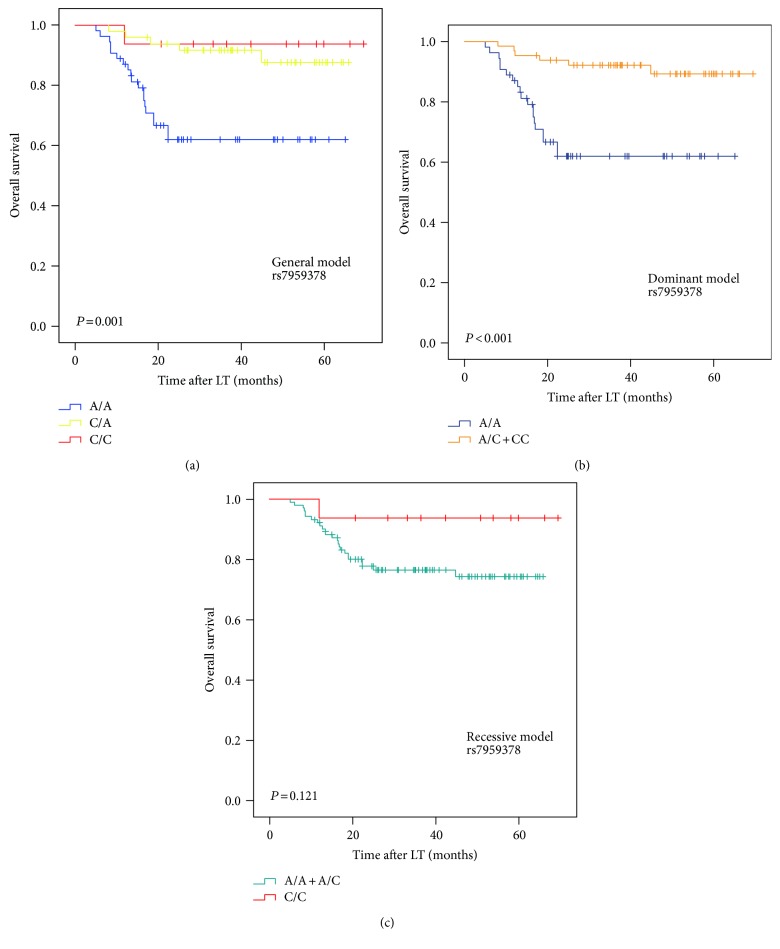
The Kaplan-Meier survival curves for overall survival of the HCC patients stratified by rs7959378 genotypes under general model (a), dominant model (b), and recessive model (c).

We next analyzed the association between the TFCP2 SNPs and the prognosis of HCC after LT. We found that rs7959378 was significantly associated with RFS and OS in Kaplan-Meier analysis. The patients who carried the AC or CC genotype (general model: *P* = 0.002) and AC + CC genotype (dominant model: *P* = 0.003) had a significantly better RFS than those with the AA genotype (Figure 1). The patients who carried the AC or CC genotype (general model: *P* = 0.001) and AC + CC genotype (dominant model: *P* < 0.001) had a significantly better OS than those with the AA genotype (Figure 2).

By the univariate Cox regression analysis, we found that patients with the AC or CC genotype (general model) and AC + CC genotype (dominant model) had a significantly better prognosis than those with the AA genotype (Table 3). Multivariate analysis according to the Cox regression hazard model adjusted by AFP, tumor size and tumor number, and differentiation was next performed to evaluate the independent predictive effect of rs7959378 polymorphism on RFS and OS (Table 3). The results showed that, compared with patients carrying the AA genotype, those with the AC genotype (adjusted *P* = 0.033, 0.012 for RFS and OS, resp.) and AC + CC genotype (adjusted *P* = 0.014, 0.006 for RFS and OS, resp.) had a significantly decreased risk of relapse and death. Taken together, these results imply that rs7959378 could be used as an independent prognostic marker for HCC after LT.

We next analyzed the association between TFCP2 rs7959378 and the clinicopathological features in all HCC subjects (Table 4). Compared with the rs7959378 AA genotype, the rs7959378 AC and CC genotypes appeared less frequently in HCC patients with a single tumor (*P* = 0.037). The rs7959378 CC genotype showed a trend toward lower frequency in larger tumors (*P* = 0.053). And no significant associations were observed between rs7959378 and other characteristics.

## 4. Discussions

Accumulating evidence has illustrated that host genetic factors are widely viewed as the common basis of the different outcomes of chronic liver diseases [20–22]. In this study, we examined the association of the seven SNPs from TFCP2 on the susceptibility of HCC in chronic liver disease subjects in a Chinese population and found that rs7959378 was significantly associated with the risk of HCC. Individuals carrying the rs7959378 C allele (AC or CC genotypes) have a decreased risk of HCC, compared to those with the AA genotype. In addition, our data showed that rs7959378 predicted postoperative relapse-free survival and overall survival for HCC patients after LT. To our knowledge, this is the first report of the genetic association between the TFCP2 gene and the risk of HCC.

TFCP2 overexpression is firstly detected in human HCC patients and associated with the stage and grade of the disease [13]. Then, TFCP2 was found to contribute to 5-fluorouracil resistance [14]. We found that TFCP2 could enhance the invasion of HCC via regulating fibronectin 1 [16]. Additionally, TFCP2 has also been identified as an important determinant of multiple cancers [23, 24]. Polymorphisms in genes, including exons, introns, and untranslated regions, have been shown to affect the processing of mRNAs as well as their regulatory effects and expressions, thus affecting the development and prognosis of different cancers [25, 26]. No studies have specifically addressed the role of TFCP2 polymorphisms in HCC so far. The result presented here showed that subjects with TFCP2 rs7959378 C allele and CC genotype had decreased risk of HCC compared with those with AA genotype after adjusting for noncomparable factors. And those patients with CC genotype also had less probability of multiple tumors.

Our results also indicated that TFCP2 rs7959378 was significantly associated with RFS and OS of HCC patients after LT. The patients with the CC and AC + CC genotypes had a significantly decreased risk of relapse and death compared with those carrying the AA genotype. Further multivariate analysis combined the Cox regression hazard model analysis confirmed rs7959378 as an independent prognostic factor. rs7959378 had a significant association with the number of tumor lesions. Based on our findings, TFCP2 rs7959378 might be used to predict which HCC patients are at risk of poor clinical outcomes in the future.

In conclusion, we report an association between TFCP2 rs7959378 and the risk of HCC and prognosis of HCC after LT, which is independent of other known risk factors. These data highlight the importance of understanding the roles of TFCP2 genetic polymorphisms in HCC pathogenesis, at least in a Chinese population. These findings suggest that TFCP2 rs7959378 could potentially be included in a multifactorial risk assessment and also used as a prognostic predictor for HCC patients who underwent LT.

## Figures and Tables

**Table 1:** Clinical data for the non-HCC and HCC groups.

Variable	Non-HCC (*n* = 200)	HCC (*n* = 119)	*P* value
Age (years), *n* (%)			0.155
≤50	128 (64)	66 (56)	
>50	72 (36)	53 (44)	

Gender			*0.008*
Male, *n* (%)	163 (82)	110 (92)	
Female, *n* (%)	37 (19)	9 (8)	

BMI (kg/m^2^)^∗^	23.2 ± 6.4	24.2 ± 8.5	0.299

Etiology of liver diseases, *n* (%)			0.164
HBV	151 (76)	98 (82)	
Others	49 (24)	21 (18)	

Child score^∗^	9.5 ± 2.1	7.5 ± 2.1	*<0.001*

MELD score^∗^	21.2 ± 11.8	12.1 ± 5.7	*<0.001*

BMI, body mass index; HBV, hepatitis B virus; HCC, hepatocellular carcinoma; MELD, model for end-stage liver disease. ^∗^The data were presented as mean ± SD. Values in italics indicate significance.

**Table 2:** Genotype distributions of the 7 SNPs in non-HCC and HCC groups.

	Genotype	Non-HCC	HCC	OR	*P*	OR^∗^	*P* ^∗^
rs7959378	AA	60 (30)	54 (45)	1 (ref)		1 (ref)	
	AC	94 (47)	49 (41)	0.58 (0.35–0.96)	*0.034*	0.64 (0.36–1.16)	0.141
	CC	46 (23)	16 (14)	0.39 (0.20–0.76)	*0.006*	0.43 (0.20–0.92)	*0.030*
	AC + CC	140 (70)	65 (55)	0.52 (0.32–0.83)	*0.006*	0.57 (0.33–0.98)	*0.044*
	A allele	214 (53)	157 (66)	1 (ref)		1 (ref)	
	C allele	186 (47)	81 (34)	0.59 (0.43–0.83)	*0.002*	0.63 (0.43–0.93)	*0.018*

rs11169736	GG	127 (63)	76 (64)	1 (ref)		1 (ref)	
	GT	66 (33)	41 (34)	1.04 (0.64–1.68)	0.879	0.98 (0.56–1.72)	0.954
	TT	7 (4)	2 (2)	0.48 (0.10–2.36)	0.364	0.25 (0.05–1.35)	0.108
	GT + TT	73 (37)	43 (36)	0.98 (0.61–1.58)	0.948	0.88 (0.51–1.51)	0.643
	G allele	320 (80)	193 (81)	1 (ref)		1 (ref)	
	T allele	80 (20)	45 (19)	0.93 (0.62–1.40)	0.737	0.80 (0.51–1.27)	0.351

rs1056897	AA	8 (4)	2 (2)	1 (ref)		1 (ref)	
	AG	69 (35)	39 (33)	2.26 (0.46–11.2)	0.317	3.79 (0.71–20.3)	0.121
	GG	123 (61)	78 (65)	2.54 (0.53–12.3)	0.247	4.49 (0.85–23.6)	0.076
	AA + AG	192 (96)	117 (98)	2.44 (0.51–11.7)	0.265	4.20 (0.81–21.8)	0.087
	A allele	85 (21)	43 (18)	1 (ref)		1 (ref)	
	G allele	315 (79)	195 (82)	1.22 (0.81–1.84)	0.332	1.40 (0.89–2.23)	0.147

rs11169735	CC	115 (58)	78 (65)	1 (ref)		1 (ref)	
	CT	70 (35)	38 (32)	0.80 (0.49–1.30)	0.372	0.94 (0.54–1.64)	0.822
	TT	15 (7)	3 (3)	0.30 (0.08–1.05)	0.060	0.63 (0.16–2.51)	0.514
	CT + TT	85 (42)	41 (35)	0.71 (0.44–1.14)	0.156	0.90 (0.52–1.54)	0.698
	C allele	300 (75)	194 (81)	1 (ref)		1 (ref)	
	T allele	100 (25)	44 (19)	0.68 (0.46–1.01)	0.058	0.88 (0.56–1.39)	0.584

rs12820966	AA	130 (65)	79 (66)	1 (ref)		1 (ref)	
	AC	62 (31)	38 (32)	1.01 (0.62–1.65)	0.973	0.95 (0.54–1.68)	0.864
	CC	8 (4)	2 (2)	0.41 (0.09–1.99)	0.269	0.25 (0.05–1.31)	0.101
	AC + CC	70 (35)	40 (34)	0.94 (0.58–1.52)	0.801	0.84 (0.49–1.46)	0.542
	A allele	322 (80)	196 (82)	1 (ref)		1 (ref)	
	C allele	78 (20)	42 (18)	0.89 (0.58–1.34)	0.563	0.77 (0.48–1.23)	0.273

rs10876135	GG	129 (64)	91 (76)	1 (ref)		1 (ref)	
	GA	66 (33)	27 (23)	0.28 (0.03–2.47)	0.254	0.53 (0.05–5.42)	0.591
	AA	5 (3)	1 (1)	0.58 (0.34–0.97)	*0.041*	0.69 (0.39–1.25)	0.223
	GA + AA	71 (36)	28 (24)	0.56 (0.34–0.93)	*0.026*	0.69 (0.38–1.22)	0.199
	G allele	324 (81)	209 (88)	1 (ref)		1 (ref)	
	A allele	76 (19)	29 (12)	0.59 (0.37–0.94)	*0.026*	0.72 (0.43–1.21)	0.214

rs10099	CC	78 (39)	56 (47)	1 (ref)		1 (ref)	
	CT	87 (44)	51 (43)	0.82 (0.50–1.33)	0.415	1.0 (0.56–1.76)	0.986
	TT	35 (17)	12 (10)	0.48 (0.23–1.00)	0.050	0.49 (0.22–1.10)	0.083
	CT + TT	122 (61)	63 (53)	0.72 (0.46–1.14)	0.159	0.83 (0.49–1.40)	0.479
	C allele	243 (61)	163 (68)	1 (ref)		1 (ref)	
	T allele	157 (39)	75 (32)	0.71 (0.51–1.0)	0.050	0.74 (0.51–1.10)	0.132

^∗^ *P* value or odds ratio after adjusting for gender, MELD score, and Child score. OR, odds ratio. Values in italics indicate significance.

**Table 3:** Association between the TFCP2 rs7959378 genotype and survival in HCC patients after LT.

rs7959378	RFS				OS			
	HR	*P*	HR^∗^	*P* ^∗^	HR	*P*	HR^∗^	*P* ^∗^
General		*0.014*		*0.041*		*0.003*		*0.021*
AA	1 (ref)		1 (ref)				1 (ref)	
AC	0.46 (0.24–0.91)	*0.026*	0.46 (0.23–0.94)	*0.033*	0.22 (0.08–0.59)	*0.003*	0.27 (0.10–0.75)	*0.012*
CC	0.19 (0.05–0.81)	*0.025*	0.25 (0.06–1.14)	0.073	0.13 (0.02–1.00)	0.050	0.16 (0.02–1.23)	0.123

Dominant								
AA	1 (ref)		1 (ref)		1 (ref)		1 (ref)	
AC + CC	0.39 (0.20–0.75)	*0.005*	0.42 (0.21–0.84)	*0.014*	0.20 (0.08–0.50)	*0.001*	0.25 (0.08–0.59)	*0.006*

Recessive								
AA + AC	1 (ref)		1 (ref)		1 (ref)		1 (ref)	
CC	0.27 (0.07–1.13)	0.073	0.39 (0.09–1.66)	0.201	0.23 (0.03–1.73)	0.155	0.34 (0.05–2.60)	0.299

^∗^Adjusted by AFP, tumor size and tumor number, and differentiation. RFS, relapse-free survival, OS, overall survival. HR, hazard ratio. Values in italics indicate significance.

**Table 4:** Association between the rs7959378 genotypes and the clinical features of HCC patients.

Features	rs7959378A>C genotypes *n* (%)			*P*
	AA	AC	CC	
AFP				0.474
≤400	41 (49)	33 (39)	10 (12)	
>400	13 (37)	16 (46)	6 (17)	

Tumor number				*0.037*
Single	27 (37)	33 (45)	13 (18)	
Multiple	27 (59)	16 (35)	3 (6)	

Tumor size				
≤5 cm	32 (39)	37 (45)	14 (16)	0.053
>5 cm	22 (61)	12 (33)	2 (6)	

Differentiation				0.622
Poor	15 (40)	18 (47)	5 (13)	
Mod./well	39 (48)	31 (38)	11 (14)	

AFP, *α*-fetoprotein. Value in italics indicates significance.
